# Supplementary material for: Identification and Characterization of Cancer-Associated Fibroblast Subpopulations in Lung Adenocarcinoma
Source: Cancers (Basel). 2022 Jul 18;14(14):3486. doi: 10.3390/cancers14143486 (PMC9324153; doi:10.3390/cancers14143486)
Supplement: Supplementary file 1 [file cancers-14-03486-s001.zip › cancers-1732991 - Supplementary Figures.pdf]

cancers MDPI

## Supplementary Figures

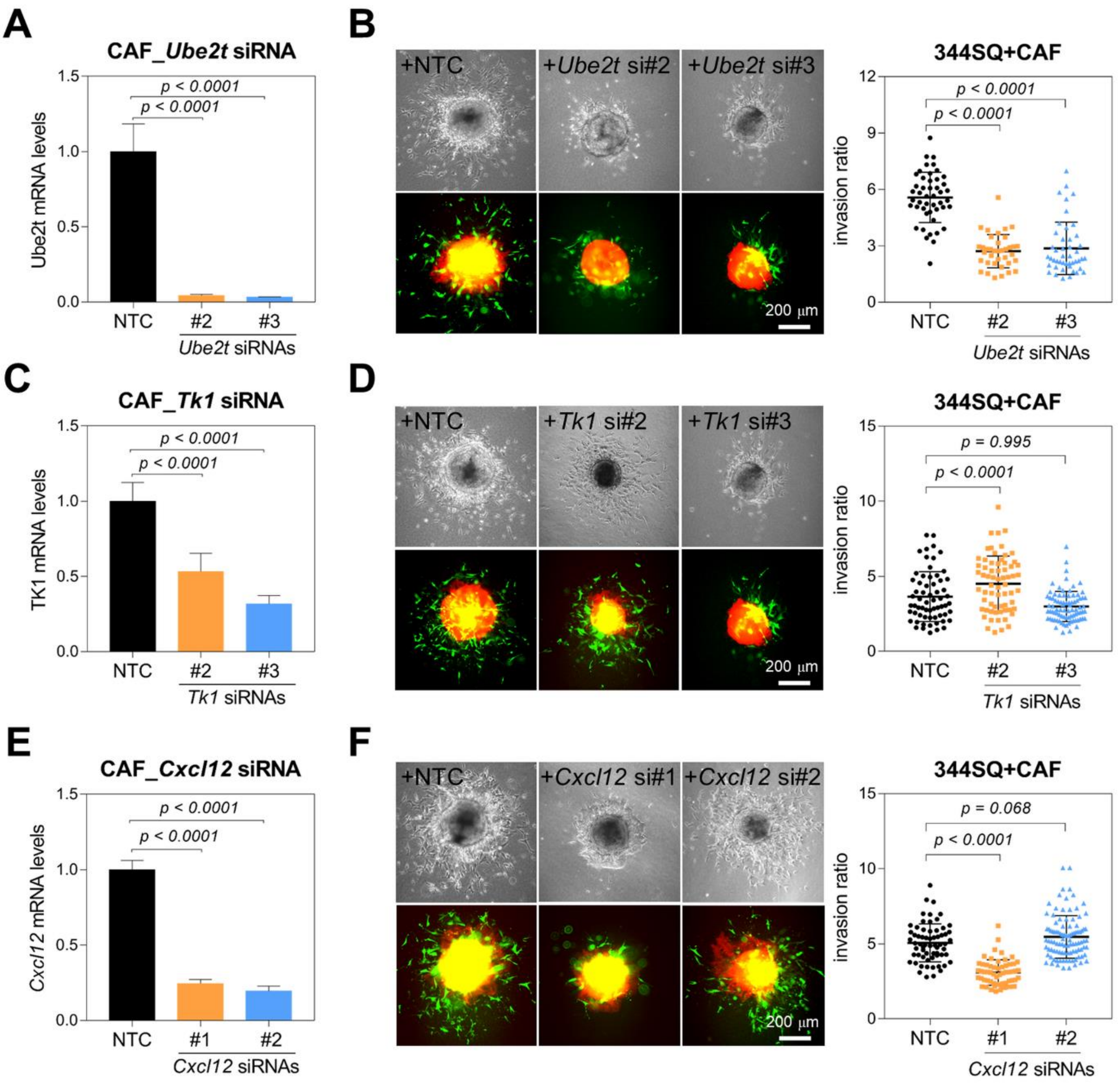


**Figure S1.** Effects of Branch 2 markers on CAF-induced invasion of cancer cells. (**A**,**C**,**E**) qRT-PCR analysis of *Ube2t*, *Tk1*, and *Cxcl12* expression in murine CAFs transfected with non-targeting control (NTC) or Branch 2 markers-specific siRNAs. Expression levels were normalized to the *Rpl32* mRNA level, and values relative to that of the NTCs (set at 1.0) are presented. *p*, two-tailed Student's *t*-test. Data are presented as the mean + SD (n = 3). (**B**,**D**,**F**) Spheroid invasion assay in 344SQ cells cocultured with CAFs transfected with NTC or Branch 2 markers-specific siRNAs. 344SQ cells were labeled with mCherry (red fluorescence), and CAFs were labeled with GFP (green fluorescence). Spheroids made from hanging-drop cultures were seeded on collagen gels and cultured for two days. Spheroid invasion ratios (ratio of whole-cell area to central spheroid area) were measured using ImageJ. Mean ± SD. *p*, two-tailed Student's *t*-test.

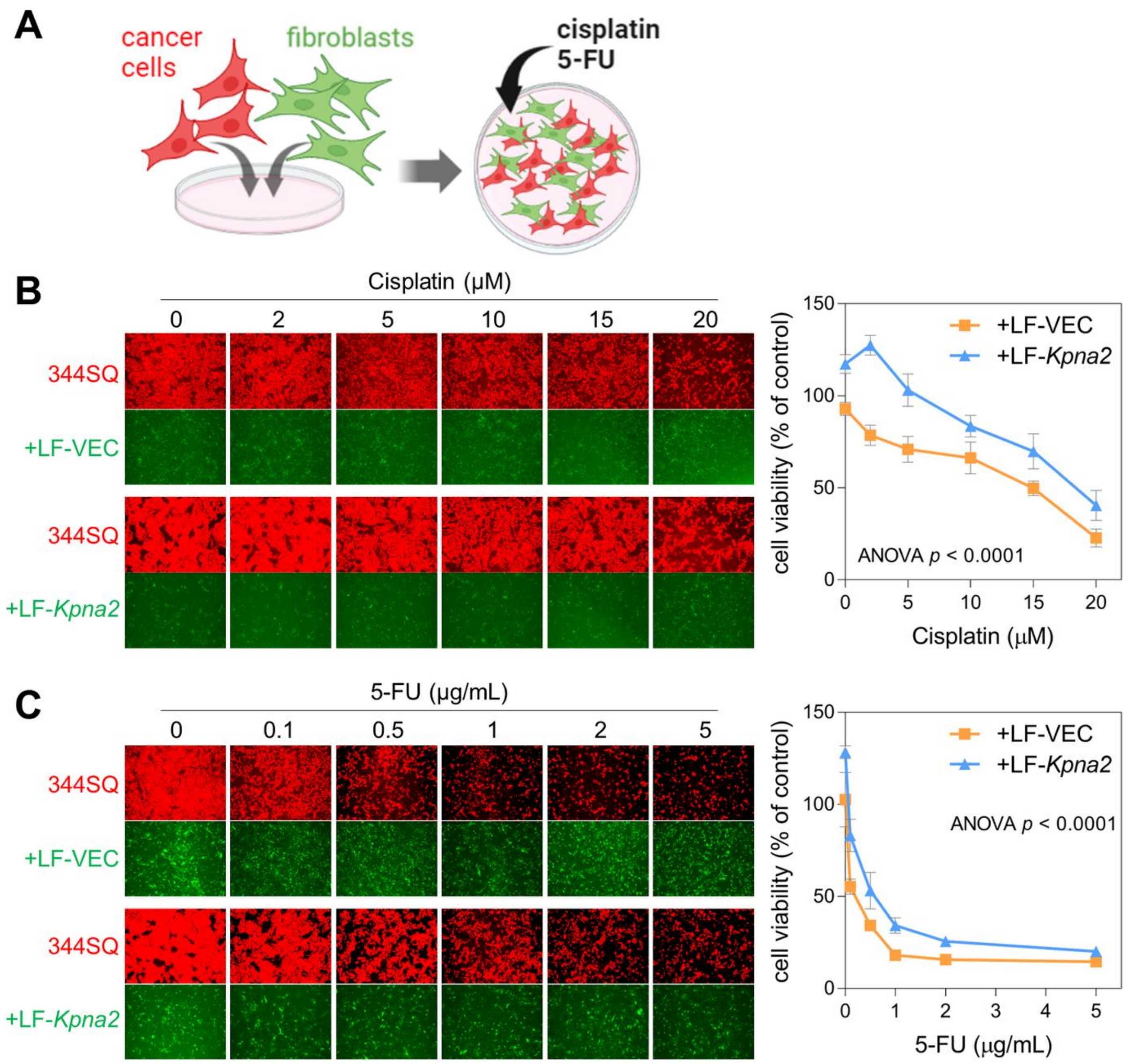


**Figure S2.** Effect of KPNA2 on CAF-induced drug resistance in cancer cells. (**A**) An illustration showing the drug treatment in coculture of 344SQ cancer cells and fibroblasts. (**B**) Cell viability of 344SQ cancer cells treated with cisplatin in the presence of LF+VEC or LF+*Kpna2*. 344SQ cells (red) were cocultured with LF+VEC or LF+*Kpna2* (green) and then treated with cisplatin (0-20 μM). After 48 h, 344SQ cells and LFs were photographed under a florescence microscope (left images), and density of 344SQ cancer cells was measured using Image J. Mean ± SD (n = 6). *p*, two-way ANOVA. (**C**) Cell viability of 344SQ cancer cells treated with 5-fluorouracil (5-FU) in the presence of LF+VEC or LF+*Kpna2*. 344SQ cells (red) were cocultured with LF+VEC or LF+*Kpna2* (green) and then treated with 5-FU (0-5 μg/mL). After 48 h, 344SQ cells and LFs were photographed under a florescence microscope (left images), and density of 344SQ cancer cells was measured using Image J. Mean ± SD (n = 6). *p*, two-way ANOVA.

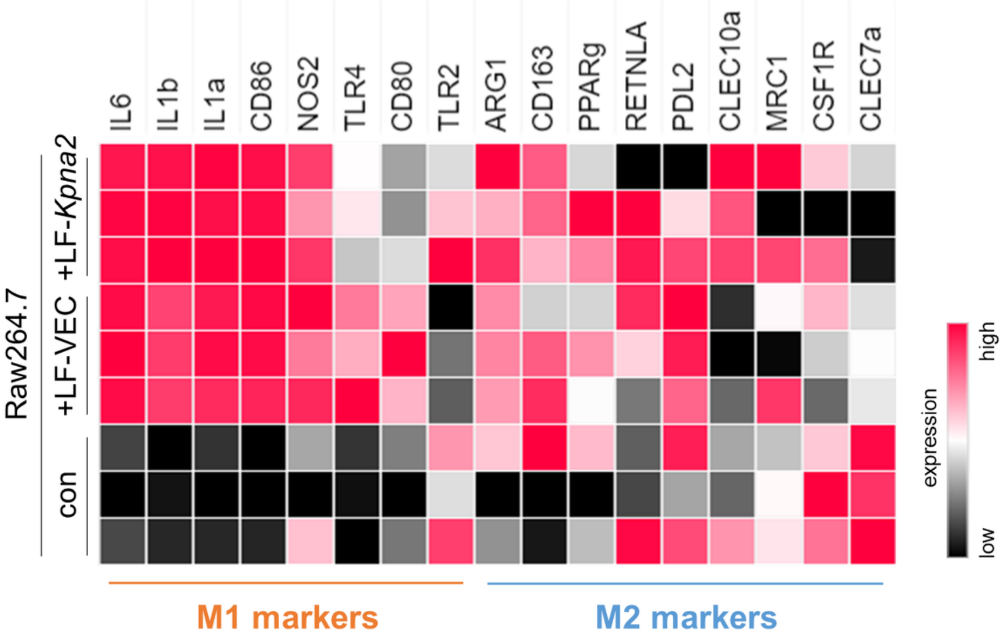


**Figure S3.** Effect of KPNA2 on CAF-induced macrophage differentiation. qRT-PCR analysis of M1- and M2-macrophage markers in Raw264.7 murine macrophages treated with conditioned media from LF+VEC or LF+*Kpna2* for 24 h. Expression levels were normalized to the *Rpl32* mRNA level and were visualized with a heatmap.

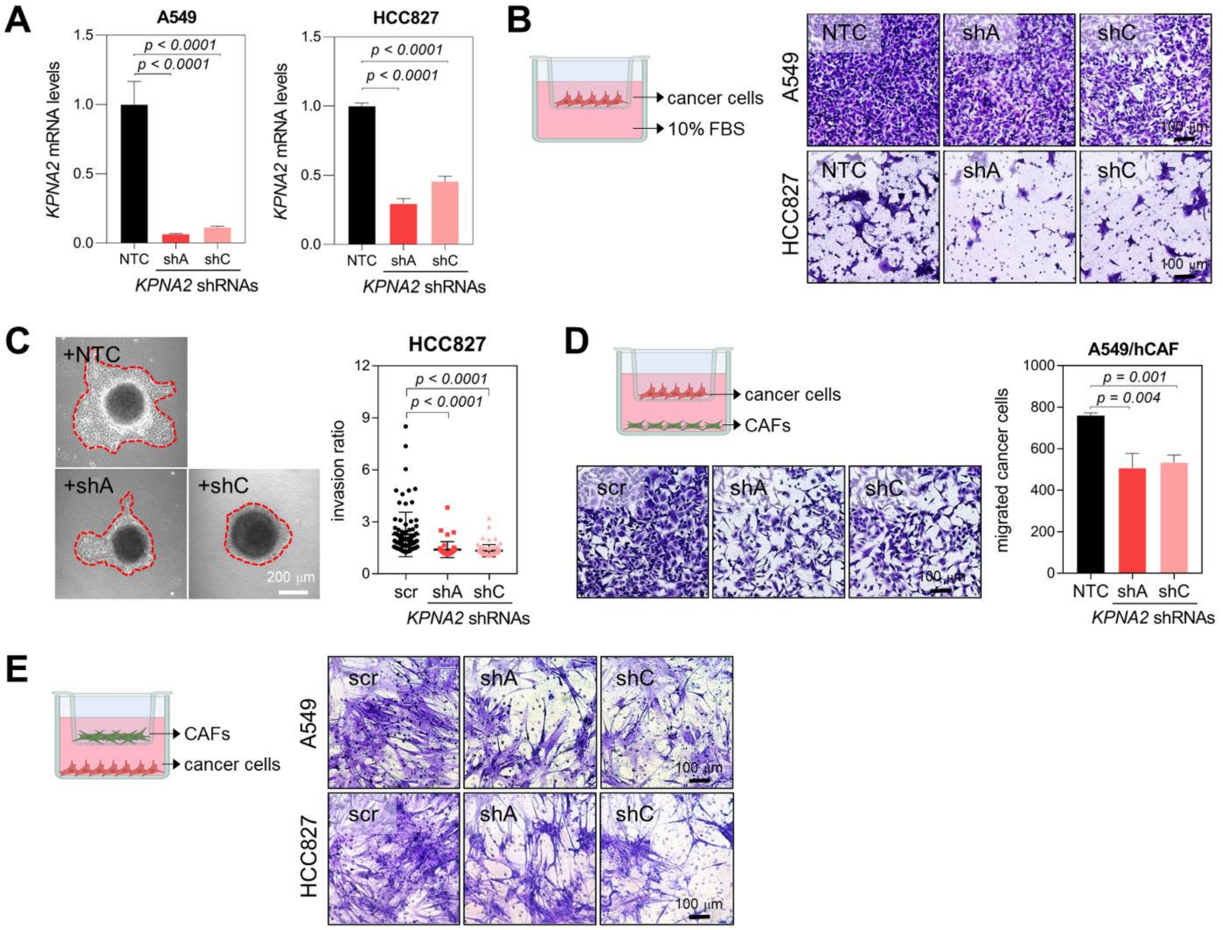


**Figure S4.** Effect of KPNA2 on cancer cell migration and invasion. (**A**) qRT-PCR analysis of *KPNA2* expression in human lung cancer cells (A549 and HCC827) transduced with nontargeting control

(NTC) or *KPNA2* shRNAs (shA and shC). Expression levels were normalized to the *RPL32* mRNA level, and values relative to that of the NTCs (set at 1.0) are presented. *p*, two-tailed Student's *t*-test. Data are presented as the mean + SD (n = 3). (**B**) Transwell migration assay in control (NTC) or *KPNA2*-knockdown (shA and shC) lung cancer cells. Cancer cells were seeded in the inserts, and complete medium with 10% FBS was added to the bottom wells. After 24 h, migrated cancer cells were photographed. (**C**) Spheroid invasion assay in control (NTC) or *KPNA2*-knockdown (shA and shC) HCC827 cells. Spheroids of cancer cells made from hanging-drop cultures were seeded on collagen gels and cultured for 7 days. Spheroid invasion ratios (ratio of whole-cell area to central spheroid area) were measured using ImageJ. Mean ± SD. *p*, two-tailed Student's *t*-test. (**D**) Transwell migration assay in A549 cells cocultured with human CAFs. Control (NTC) or *KPNA2*-knockdown (shA and shC) A549 cells were seeded in the inserts, and CAFs were seeded in the bottom wells. After 24 h, migrated A549 cells were photographed and counted. Mean + SD (n = 3). *p*, two-tailed Student's *t*-test. (**E**) Transwell migration assay in human CAFs co-cultured with lung cancer cells. Human CAFs were seeded in the inserts, and control (NTC) or *KPNA2*-knockdown (shA and shC) human lung cancer cells (A549 and HCC827) were seeded in the bottom wells. After 24 h, migrated CAFs were photographed and counted. Mean + SD (n = 3). *p*, two-tailed Student's *t*-test.

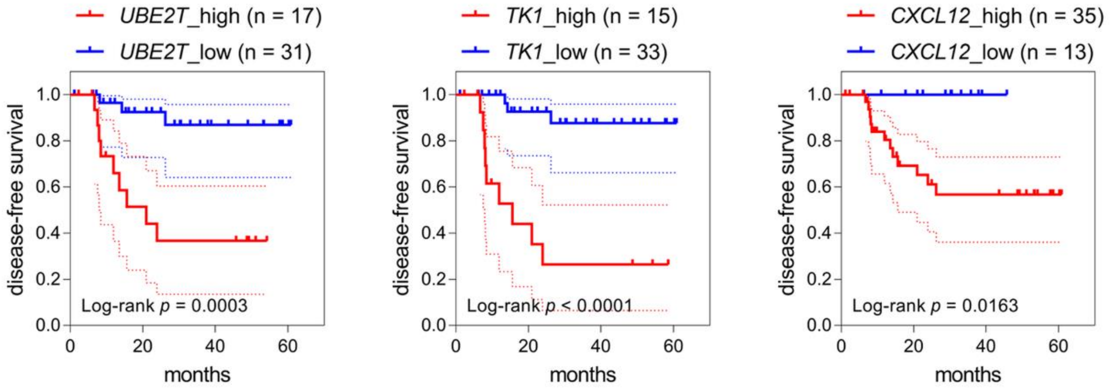


**Figure S5.** Effects of Branch 2 markers on the patient survival in lung adenocarcinoma. Kaplan-Meier plots showing the disease-free survival of patients with lung adenocarcinoma. Patients were divided into two groups (high and low) based on their *UBE2T*, *TK1*, and *CXCL12* mRNA expression levels. Dotted lines represent 95% confidence intervals. *p*-Values were determined via the log-rank test.
